# The Effect of MicroRNA-101 on Angiogenesis of Human Umbilical Vein Endothelial Cells during Hypoxia and in Mice with Myocardial Infarction

**DOI:** 10.1155/2020/5426971

**Published:** 2020-09-03

**Authors:** Jun Pang, Liwen Ye, Qingwei Chen, Jian Wang, Xixi Yang, Wuyang He, Lan Hao

**Affiliations:** ^1^Department of Geriatrics, The Second Affiliated Hospital of Chongqing Medical University, Chongqing 400010, China; ^2^Chongqing Key Laboratory of Ultrasound Molecular Imaging, Ultrasound Department of the Second Affiliated Hospital of Chongqing Medical University, Chongqing 400010, China; ^3^Institute of Anorectal Diseases, North Sichuan Medical College, Nanchong 637000, China; ^4^Department of Anesthesiology, The Second Clinical College of North Sichuan Medical College, Nanchong Central Hospital, Nanchong 637000, China; ^5^State Key Laboratory of Ultrasound Engineering in Medicine Co-Founded by Chongqing and the Ministry of Science and Technology, Chongqing Medical University, Chongqing 400010, China

## Abstract

**Background:**

Previous studies showed that recanalization and angiogenesis within the infarct region are of vital importance to the survival of myocardial cells during the treatment of acute myocardial infarction (AMI).

**Methods:**

In this study, EdU cell proliferation assay, Transwell assay, scratch wound assay, and tube formation assay were used. Twelve bioinformatics analysis packages were used to predict the target genes of miR-101. Target genes were verified by luciferase reporter generation and assay, fluorescent quantitative PCR, and western blotting. Animal model and treatments were detected by M-mode echocardiography and immunofluorescent staining of CD31, Ki67, and *α*-SMA.

**Results:**

AgomiR-101 significantly enhanced HUVEC proliferation, migration, and tube formation. A double-luciferase reporter assay revealed that the hsa-miR-101 mimic attenuated the activity of the EIF4E3′-UTR-wt type plasmid by 36%. The expression levels of HIF-1*α* and VEGF-A in the scrambled RNA group were significantly lower than those in the EIF4E3 siRNA and agomiR-101 groups. The left ventricular ejection fraction of the AMI+Adv-miR-101 group was significantly higher than that of the AMI+Adv-null and Sham+Adv-null groups. The proliferation of vessel cells in the peripheral infarcted myocardium was higher in the AMI+Adv-miR-101 group than that in the AMI+Adv-null and Sham+Adv-null groups.

**Conclusion:**

MiR-101 can promote angiogenesis in the region surrounding the myocardial infarction.

## 1. Introduction

Heart failure, caused by acute myocardial infarction (AMI), is a major cause of mortality worldwide. More than three million people suffer from acute ST-segment elevation myocardial infarction every year [1]. In recent years, the incidence of myocardial infarction has increased [2]; thus, it is necessary to explore the pathogenesis of AMI.

Previous studies have shown that recanalization and angiogenesis in the infarct area are vital to the survival of myocardial cells during the treatment of AMI. At present, therapeutic angiogenesis can effectively establish collateral circulation, improve blood supply to the myocardial infarct area, increase the viability of myocardial cells, and improve the condition of myocardial infarction [3]. Some studies [4–7] have shown that multiple angiogenesis stimulating factors are involved in angiogenesis.

MicroRNAs (miRNAs) are small, highly conserved, endogenous, single-stranded RNA molecules of approximately 19–25 nucleotides that negatively regulate protein expression in various organisms [8]. A number of studies demonstrated that miRNAs have regulatory roles in cardiovascular diseases [9]. In particular, miR-101 functions in the repair and therapeutic regeneration of cardiovascular diseases [10].

The effect of miR-101 on the formation of angiogenesis after AMI has yet to be reported. In the current study, AMI cell and animal models were used to examine whether miR-101 plays an important role in the proliferation, migration, and angiogenesis of vascular endothelial cells, as well as the recovery of cardiac function. Moreover, to identify new therapeutic tactics for the treatment of AMI, the targets and signaling pathways of miR-101 in angiogenesis were examined.

## 2. Materials and Methods

### 2.1. Cell Culture

Human umbilical vein endothelial cells (HUVECs) were cultivated in Dulbecco's modified Eagle medium (DMEM), containing 1% fetal bovine serum (FBS) and penicillin/streptomycin, and were maintained at 37°C in a 5% CO_2_ incubator.

### 2.2. EdU Cell Proliferation Assay

When the cell fusion reached 80%, the cells were divided into three groups and agomiR-101, antagomiR-101, and scrambled RNA were added to the culture medium at a final concentration of 200 nM. The culture medium also contained 100 *μ*M CoCl_2_ and 10% FBS. HUVEC proliferation was detected 48 h after cultivation using an EdU assay. A fluorescent microscope was used to observe the samples and obtain images.

### 2.3. Scratch Wound Assay

Approximately 3 × 10^5^ HUVECs were inoculated on a 6-well plate. After the HUVECs reached 70% confluence, they were transfected with agomiR-101, antagomiR-101, or scrambled RNA in 100 *μ*M CoCl_2_. At 48 h post-transfection, three parallel lines were drawn on the back of the 6-well plate using a marker pen and the cell layer was scratched perpendicular to the transverse lines using a 100 *μ*l Finnpipette. The cells were then washed three times with 0.01 M phosphate-buffered saline (PBS) and cultured in DMEM containing 1% fetal calf serum at 37°C and 5% CO_2_. The intersection points between the scratches and the marked lines were photographed after 24 h and scratch wound healing was determined.

### 2.4. Transwell Assay

After starvation for 24 h, the cell suspension was collected, centrifuged, and washed twice. A 400 *μ*l aliquot of cell suspension was then added to the upper chamber of a Transwell plate. NIH3T3 culture solution (600 *μ*l) was added to the lower chamber as the chemotaxis liquid. The culture plate was incubated for 48 h at 37°C in a 5% CO_2_ incubator. The cells on the filter membrane of the Transwell insert were carefully removed and subsequently fixed with methanol for 5 min and rinsed three times with PBS for 3 min per rinse. Hematoxylin and eosin staining was performed, and the numbers of penetrating cells were determined in five randomly selected high power fields per filter membrane.

### 2.5. Tube Formation Assay

Precooled Matrigel (300 *μ*l/well) was added to a 24-well plate and incubated at 37°C for 30 min to solidify the Matrigel. HUVECs were transfected with agomiR-101, antagomiR-101, or scrambled RNA at a final concentration of 200 nM for 48 h in 100 *μ*M CoCl_2_ and then inoculated on the surface of the solidified Matrigel. After mixing, the sample was incubated for 6 h at 37°C and 5% CO_2_. Tube formation was observed, and the circumferences of the tubes were measured in five high power fields.

### 2.6. Bioinformatics Analyses

Twelve bioinformatics analysis packages were used to predict the target genes of miR-101, namely, miRanda, miRSystem, miRDB, RNAhybrid, miRMap, TargetScan, miRNAsMap, miRWalk, PicTar, PITA, DIANA-microT, and RNA22. Based on the sequence of mature miRNA-101 (UACAGUACUGUGAUAACUGAA), the gene encoding eukaryotic translation initiation factor E3 (EIF4E3) was predicted as a target.

### 2.7. Luciferase Reporter Generation and Assay

#### 2.7.1. Construction of the Luciferase Reporter Plasmids

Luciferase reporter plasmids harboring the wild-type 3′-UTR of *EIF4E3*, containing the predicted miR-101-binding site (EIF4E3′-UTR-wt; GUACUGU), or a mutated version of the UTR containing a point mutation in the miR-101 recognition site (EIF4E3′-UTR-muta; GUACUGU), were constructed.

#### 2.7.2. Plasmid Transfection

Human 293 T cells were inoculated on a 48-well plate and incubated for 24 h at 37°C and 5% CO_2_ until the cell density reached 80%. Lipofectamine 3000 (1 *μ*l) was added to 25 *μ*l of Opti-MEM, and the mixture was stored at room temperature for 5 min. Plasmid (0.2 *μ*g) was added to another 25 *μ*l aliquot of Opti-MEM, and the mixture was stored at 25°C for 5 min. Additionally, agomiR-101 or scrambled RNA (7.5 pmol) was added to 25 *μ*l of Opti-MEM, and the mixture was incubated at room temperature for 5 min. The transfection reagent, plasmid, and agomiR-101 or scrambled RNA solutions were mixed and incubated at room temperature for 20 min. Culture medium (50 *μ*l/well) and the transfection complex (50 *μ*l/well) were added to each well of the 48-well plate (three wells per group), and the cells were incubated at 37°C and 5% CO_2_. The transfection mixture was subsequently replaced with 200 *μ*l of fresh medium and the cells were incubated at 37°C and 5% CO_2_ for a further 48 h.

#### 2.7.3. Luciferase Assay

A 200 *μ*l aliquot of cell lysis buffer was added to each well of a 48-well plate and incubated at room temperature for 8 min. The sample was then centrifuged at 4°C and 10000 g for 5 min, and the lysis supernatant was collected. A 20 *μ*l aliquot of the lysis supernatant and a 100 *μ*l aliquot of firefly luciferase working liquid were added to each well of a 96-well plate, and the luciferase signal was measured. As a control, a 100 *μ*l aliquot of Renilla luciferase working fluid was added to each well.

### 2.8. Fluorescent Quantitative PCR

Total RNA was extracted from HUVECs using TRIzol reagent (Takara, China) and reverse transcribed using the miRNA reversible transcription PCR kit (Promega, USA). The fluorescent quantitative PCR assay was performed using the following primers: EIF4E3, Forward: TGCAAAGGGTGGCGTATGGAAG, Reverse: TCTTCTCGGTCCCGAACACTGA; EIF4E1, Forward: ATGCCTGGCTGTGACTACTCAC, Reverse: GAGGTCACTTCGTCTCTGCTGT; VEGF-A, Forward: TTGCCTTGCTGCTCTACCTCCA, Reverse: GATGGCAGTAGCTGCGCTGATA; HIF-1*α*, Forward: TTGCCTTGCTGCTCTACCTCCA, Reverse: GATGGCAGTAGCTGCGCTGATA; and GAPDH, Forward: GTCTCCTCTGACTTCAACAGCG, Reverse: ACCACCCTGTTGCTGTAGCCAA.

A fluorescent qRT-PCR detection system (Bio-Rad, USA) was used for amplification, and a fluorescent quantitation PCR kit (Promega, USA) was used for detection of the target genes. The reactions were performed using SYBR green mix, cDNA, each primer, and RNase-free water. The cycling conditions were as follows: denaturation at 95°C for 10 min, followed by 40 cycles at 95°C for 2 s and 60°C for 20 s, and then a final extension at 70°C for 10 s. The expression of *β*-actin was used as an internal reference, and the relative expression of miR-101 was calculated using the 2^-*ΔΔ*^ Ct method.

### 2.9. Western Blotting

Protein was extracted from HUVECs using RIPA Lysis Buffer (Millipore, USA), and protein concentration was measured using the BCA Protein Quantitative Kit (Beyotime, China). The proteins were separated by SDS-PAGE and then transferred to a PVDF membrane. The following primary antibodies were used: rabbit monoclonal anti-HIF-1*α* (ab179483; Abcam, UK), rabbit polyclonal anti-VEGF-A (ab46154; Abcam), rabbit polyclonal anti-EIF4E3 (17282-1-AP; Proteintech, USA), rabbit polyclonal anti-EIF4E1 (17282-1-AP; Cell Signaling Technology, USA), and rabbit polyclonal anti-GAPDH (10494-1-AP; Proteintech). After incubation with the primary antibodies, the membrane was incubated with horseradish peroxidase-labeled goat-anti-rabbit IgG (ZB-2301, ZSGB-Bio, China). The immunoproteins were detected using the Hypersensitivity ECL Luminescence Kit (Beyotime), and band densities were quantified using Quantity One v4.6.2 (Bio-Rad).

### 2.10. Animal Model and Treatments

Animals were used in strict accordance with the *Guide for the Care and Use of Laboratory Animals* under the approval of the Institutional Ethics Committee of Chongqing Medical University (Permit No. SCXK (Chongqing) 2007-0001) and the State Science and Technology Commission of China. The present study made use of 22 eight-week-old C57BL/6 mice, bred in the Animal Center of Chongqing Medical University. Mice were fed with a standard chow diet. The AMI model was established by ligating the anterior descending branch of the coronary artery via thoracotomy. The mice were randomly divided into the AMI+Adv-miR-101 (*n* = 10, death = 3), AMI+Adv-null (*n* = 11, death = 4), and Sham+Adv-null (*n* = 10, death = 2) groups. A viral vector containing miR-101, or a null viral vector (negative control), was injected into the left ventricular anterior wall infarction and peripheral infarction area at three points. After two weeks, the mice were euthanized by cervical dislocation. The hearts were removed and washed with saline. Paraffin and frozen sections were then prepared, and the latter were stored at -20°C.

### 2.11. Parasternal Short-Axis M-Mode Echocardiography

After 14 days of AMI, M-mode echocardiography was used to detect changes in cardiac function in the C57BL/6 mice. A Vivid E9 diasonograph (GE Healthcare, USA) was used for detection. The transducer frequency was 15 MHz, and the depth was 1.0–1.5 cm. Mice were intraperitoneally injected with 1% pentobarbital sodium at a dose of 50 mg/kg and then fixed on the operating plate in a supine position. The fur was removed from the chest, and the coupling agent was evenly coated. The ultrasonic probe was then applied carefully to the chest, and images were collected.

### 2.12. Immunofluorescent Staining of CD31, Ki67, and *α*-SMA

For immunostaining, frozen sections of the mouse hearts were removed from the -20°C freezer, thawed at room temperature for 30 min, and washed three times with 0.01 M PBS (5 min per wash). A 2% BSA solution or immunostaining sealant was dripped onto the tissue sections; thereafter, the sections were sealed at 37°C for 1 h and dried with filter paper. Subsequently, the anti-CD31, anti-*α*-SMA, and anti-Ki67 primary antibodies were diluted at a ratio of 1 : 100 with 0.1% Triton X-100 and dripped onto the tissue sections. The sections were incubated overnight at 4°C and then washed three times with 0.01 M PBS (20 min per wash). Next, the secondary antibodies were dripped into each tissue section, and the sections were bathed in water at 37°C in the dark. The samples were then washed three times with 0.01 M PBS (10 min per wash). The sections were stained with DAPI for 10 min at room temperature in the dark. An antifluorescence quenching agent was then dripped onto the samples, and cover glasses were applied. Images were obtained using a fluorescence microscope.

### 2.13. Statistical Analysis

Image-Pro Plus software was used to analyze the proliferation rate, apoptosis rate, and vessel density of endothelial cells. Statistical analyses were performed using GraphPad Prism 6.0 software. All data are presented as the mean ± standard deviation. A one-way analysis of variance (ANOVA) was used when analyzing single variables, a multiway ANOVA was used to analyze two or more variables, and between-group comparisons were performed using the Kruskal-Wallis tests. *P* < 0.05 was considered statistically significant.

## 3. Results

### 3.1. The Effect of Hypoxia on miR-101 Expression in HUVECs

**Figure 1:**
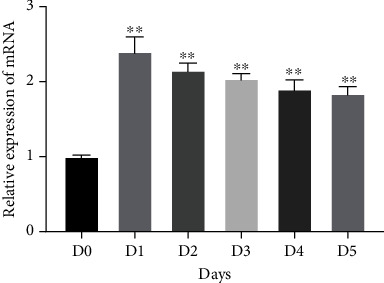
The miR-101 expressions in HUVECs with CoCl_2_ detected by qRT-PCR for 5 days (^∗∗^*P* < 0.01 vs. D0).

Quantitative RT-PCR analyses were used to examine the expression levels of miR-101 in HUVECs exposed to hypoxia for 0, 1, 2, 3, 4, or 5 days. miR-101 expression increased rapidly from the first day of exposure. On days 2–5, miR-101 expression was significantly higher than at the baseline (Figure 1).

### 3.2. The Effect of AgomiR-101 on the Proliferation, Migration, and Tube Formation of HUVECs

**Figure 2:**
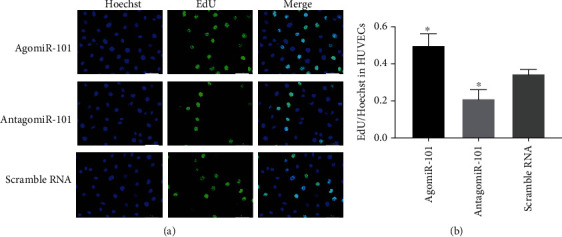
(a) Photomicrographs show the EdU cell proliferation assay of HUVECs transfected with agomiR-101, antagomiR-101, or negative control (scrambled RNA). (b) Bar graphs show the proliferation rate of HUVECs detected by EdU cell proliferation assay (^∗^*P* < 0.05 vs. scrambled RNA).

We examined the effects of agomiR-101, antagomiR-101, and a scrambled RNA (negative control) on the proliferation, migration, and tube formation of HUVECs. An EdU assay was used to determine cell proliferation. The number of EdU-labeled HUVECs in the agomiR-101 group was significantly higher than that in the scrambled RNA group. In addition, the number of EdU-labeled cells in the scrambled RNA group was significantly higher than that in the antagomiR-101 group (Figures 2(a) and 2(b)).

**Figure 3:**
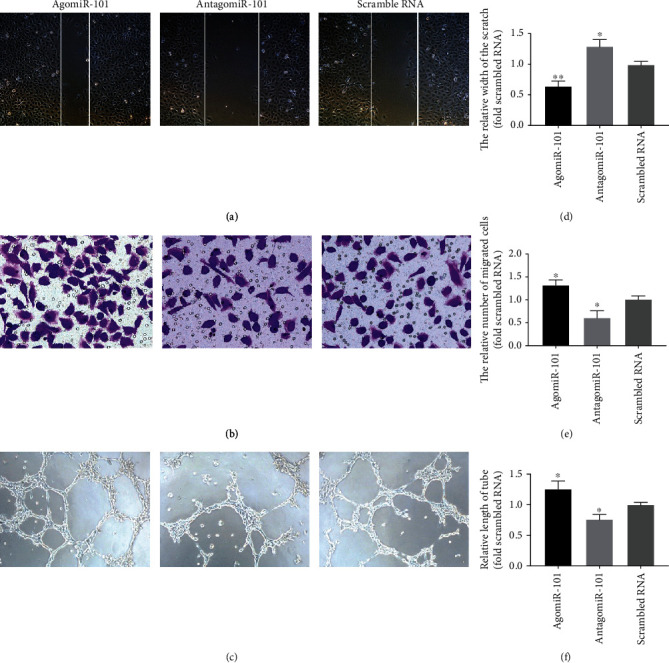
Photomicrographs (a, b, and c) show the scratch wound assay, Transwell assay, and tube formation assay of HUVECs transfected with agomiR-101, antagomiR-101, or negative control (scrambled RNA). Bar graphs (d, e, and f) show the results of scratch wound assay, Transwell assay, and tube formation assay (^∗^*P* < 0.05 vs. scrambled RNA; ^∗∗^*P* < 0.01 vs. scrambled RNA).

In the scratch wound assay, agomiR-101 and antagomiR-101 promoted and inhibited HUVEC migration during wound healing, respectively (Figures 3(a) and 3(d)). In addition, a Transwell assay showed that the number of migrating cells in the agomiR-101 group was significantly higher than that in the scrambled RNA group, and the number of migrating cells in the scrambled RNA group was significantly higher than that in the antagomiR-101 group (Figures 3(b) and 3(e)). A tube formation assay revealed that the blood vessel in the agomiR-101 group was significantly longer than in the antagomiR-101 group, and the blood vessel in the antagomiR-101 group was significantly shorter than in the scrambled RNA group (Figures 3(c) and 3(f)). Overall, the results of these experiments show that agomiR-101 significantly enhanced HUVEC proliferation, migration, and tube formation.

### 3.3. The Effect of miR-101 on *EIF4E3* Expression

**Figure 4:**
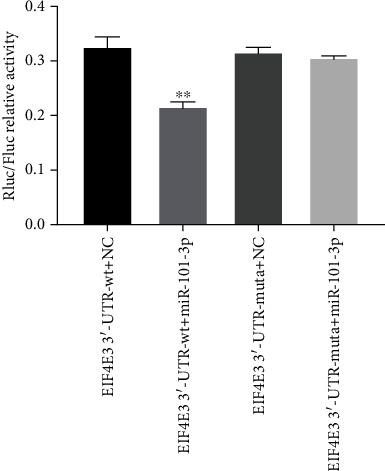
Double-luciferase reporter gene was used to detect the inhibition level of miR-101 targeting on EIF4E3 (^∗∗^*P* < 0.01 vs. EIF4E3′-UTR-wt+NC).

Various miRNA target prediction packages identified *EIF4E3* as a potential target of miR-101; thus, we examined the effects of miR-101 on *EIF4E3* expression in HUVECs. Two luciferase reporter plasmids containing the wild-type 3′-UTR of *EIF4E3* (EIF4E3′-UTR-wt) or a mutated version of the UTR containing a point mutation in the miR-101 recognition site (EIF4E3′-UTR-muta) were generated and transfected into HUVECs. The cells were also transfected with a negative control or hsa-miR-101 mimic. The results of a double-luciferase (firefly and Renilla) reporter assay revealed that the hsa-miR-101 mimic attenuated the activity of the EIF4E3′-UTR-wt type plasmid by 36%, but had no effect on the activity of the EIF4E3′-UTR-muta plasmid (Figure 4).

### 3.4. The Effects of an EIF4E3 siRNA and AgomiR-101 on the Expression Levels of EIF4E3, EIF4E1, HIF-1*α*, and VEGF-A

**Figure 5:**
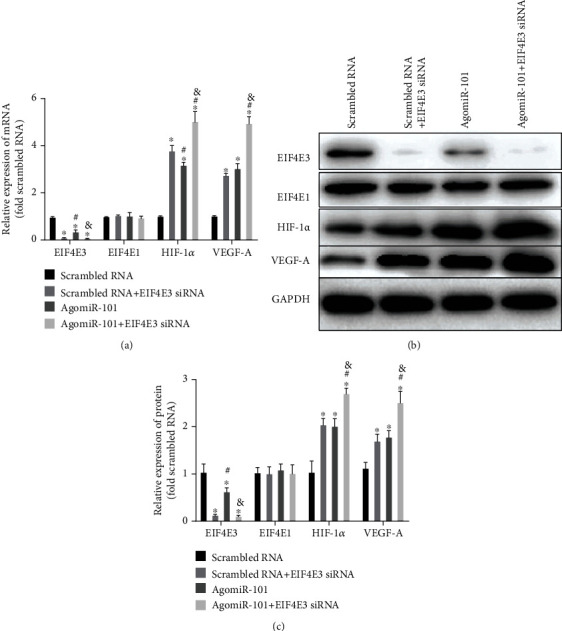
Bar graphs show the effect of scrambled RNA, scrambled RNA+EIF4E3 siRNA, agomiR-101, and agomiR-101+EIF4E3 siRNA on the mRNA (a) and protein expression (b, c) of EIF4E3, EIF4E1, HIF-1*α*, and VEGF-A (^∗^*P* < 0.01 vs. scrambled RNA; ^#^*P* < 0.01 vs. scrambled RNA+EIF4E3 siRNA; ^&^*P* < 0.01 vs. agomiR-101).

HUVECs were transfected with scrambled RNA, scrambled RNA+EIF4E3 siRNA, agomiR-101, and agomiR-101+EIF4E3 siRNA for 48 h. Subsequently, western blotting and qRT-PCR were used to detect the expression levels of EIF4E3, EIF4E1, hypoxia-inducible factor 1*α* (HIF-1*α*), and vascular endothelial growth factor A (VEGF-A) (Figures 5(a)–5(c)). The expression levels of EIF4E3 in the scrambled RNA+EIF4E3 siRNA and agomiR-101 groups were significantly lower than those in the scrambled RNA group, but they were higher than those in the agomiR-101+EIF4E3 siRNA group. However, EIF4E1 expression was not affected by agomiR-101 or the downregulation of EIF4E3. The expression levels of HIF-1*α* and VEGF-A in the scrambled RNA group were significantly lower than those in the EIF4E3 siRNA and agomiR-101 groups. There were no significant differences in HIF-1*α* and VEGF-A expression between the EIF4E3 siRNA group and the agomiR-101 groups; however, the expression level of HIF-1*α* and VEGF-A in the agomiR-101+EIF4E3 siRNA group was higher than that in the agomiR-101 group, as well as the EIF4E3 siRNA group.

### 3.5. The Effects of an AgomiR-101, Antagomir-101, and Scrambled RNA on the Expression Levels of EIF4E3, EIF4E1, HIF-1*α*, and VEGF-A

**Figure 6:**
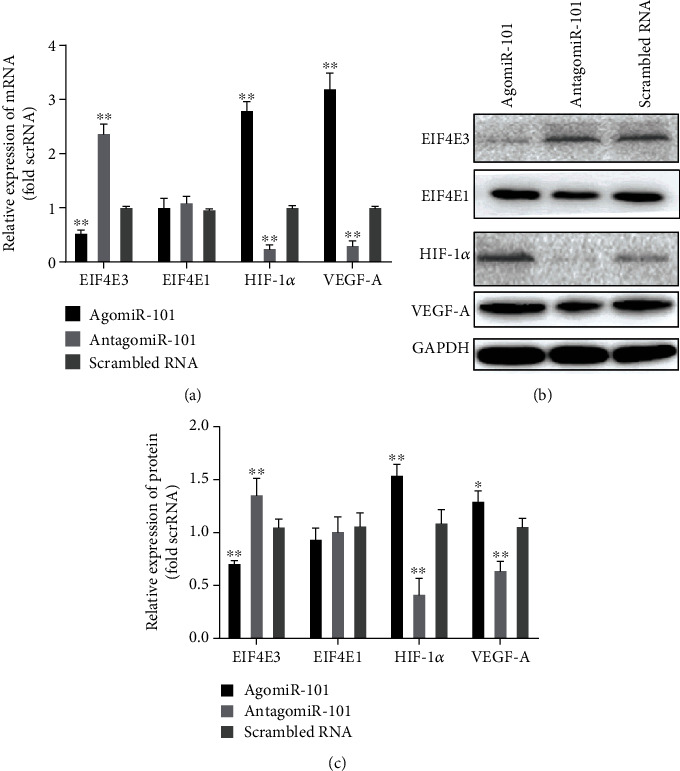
Bar graphs show the effect of agomiR-101, antagomiR-101, and scrambled RNA on the mRNA (a) and protein expression (b, c) of EIF4E3, EIF4E1, HIF-1*α*, and VEGF-A (^∗^*P* < 0.05 vs. scrambled RNA; ^∗∗^*P* < 0.01 vs. scrambled RNA).

HUVECs were transfected with agomiR-101, antagomiR-101, and scrambled RNA for 48 h. Western blotting and qRT-PCR were used to detect the expression levels of EIF4E3, EIF4E1, hypoxia-inducible factor 1*α* (HIF-1*α*), and vascular endothelial growth factor A (VEGF-A) (Figures 6(a)–6(c)). The expression levels of EIF4E3 in the agomiR-101 groups were significantly lower than those in the scrambled RNA group, but were higher than those in the antagomiR-101 group. However, EIF4E1 expression was not affected by agomiR-101 or antagomir-101. The expression levels of HIF-1*α* and VEGF-A in the scrambled RNA group were significantly lower than those in the agomiR-101 group, but were higher than those in the antagomiR-101 group.

### 3.6. The Effect of miR-101 on Cardiac Function in C57BL/6 Mice

Two weeks after the establishment of AMI or sham treatment as a control, M-mode echocardiography was used to detect the cardiac function of C57BL/6 mice that were injected at the left ventricular anterior wall infarction and peripheral infarction area, with a viral vector containing miR-101 (Adv-miR-101) gene or a null viral vector as a negative control (Adv-null). The left ventricular ejection fraction (LVEF) of the AMI+Adv-miR-101 group was significantly higher than that of the AMI+Adv-null and Sham+Adv-null groups, and the LVEF of the Sham+Adv-null group was significantly higher than that of the AMI+Adv-null group (Table 1). Similarly, the left ventricular shortening fraction of mice in the AMI+Adv-miR-101 group was significantly higher than that of mice in the AMI+Adv-null and Sham+Adv-null groups, and that of mice in the Sham+Adv-null group was significantly higher than that in the AMI+Adv-null group. Finally, the left ventricular end-systolic diameter of mice in the AMI+Adv-miR-101 group was significantly lower than that in the AMI+Adv-null and Sham+Adv-null groups. Overall, the results indicated that miR-101 improved the cardiac function of AMI mice.

### 3.7. The Effect of miR-101 on the Proliferation of Vascular Endothelial Cells in Myocardial Infarction Mice

**Figure 7:**
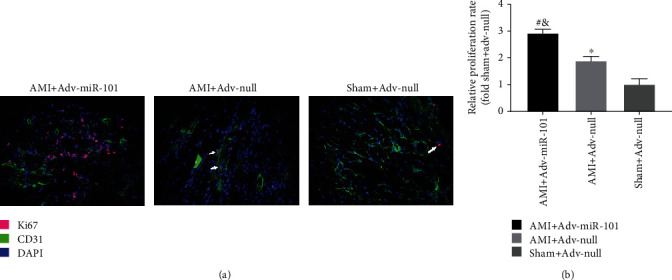
Photomicrographs (a) and bar graphs (b) show the proliferation of vascular endothelial cells of the peripheral infarcted myocardium (^∗^*P* < 0.05 vs. Sham+Adv-null; ^&^*P* < 0.01 vs. Sham+Adv-null; ^#^*P* < 0.05 vs. AMI+Adv-null).

Immunofluorescent staining was used to evaluate the proliferation of vascular endothelial cells in AMI and sham-treated C57BL/6 mice after injection of Adv-miR-101 or Adv-null for two weeks (Figures 7(a) and 7(b)). The proliferation of these cells in the peripheral infarcted myocardium was significantly higher in the AMI+Adv-miR-101 group than that in the AMI+Adv-null and Sham+Adv-null groups.

### 3.8. The Effect of miR-101 on Myocardial Vessel Density in Myocardial Infarction Mice

**Figure 8:**
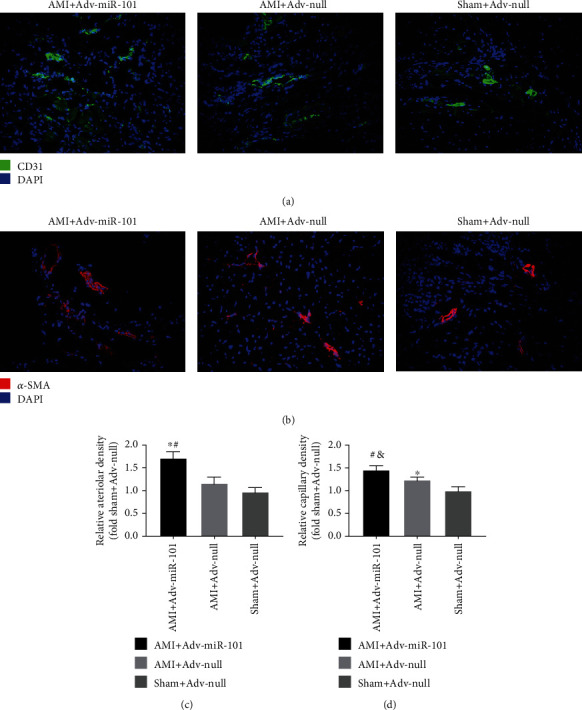
Photomicrographs (a, b) and bar graphs (c, d) show the vessel density and arteriole density of the peripheral infarcted myocardium (^∗^*P* < 0.05 vs. Sham+Adv-null; ^&^*P* < 0.01 vs. Sham+Adv-null; ^#^*P* < 0.05 vs. AMI+Adv-null).

CD31 immunofluorescent staining was used to examine the myocardial vessel densities in the AMI+Adv-miR-101, AMI+Adv-null, and Sham+Adv-null groups (Figures 8(a) and 8(c)). The vessel density of the peripheral infarcted myocardium in the AMI+Adv-miR-101 group was significantly higher than that in the AMI+Adv-null and Sham+Adv-null groups. In addition, the vessel density of the peripheral infarcted myocardium in the AMI+Adv-null group was higher than that in the Sham+Adv-null group. Immunofluorescent staining of *α*-SMA was also used to assess the density of arterioles (Figures 8(b) and 8(d)). The arteriole density of the peripheral infarcted myocardium was higher in the AMI+Adv-miR-101 group than that in the AMI+Adv-null and Sham+Adv-null groups. There were no significant differences between the arteriole densities of the peripheral infarcted myocardium in the Sham+Adv-null group and AMI+Adv-null groups.

## 4. Discussion

AMI is a clinical syndrome characterized by rupture of atheromatous plaque and acute arterial occlusion, resulting in myocardial necrosis. Recirculation of the residual blood vessels in the infarcted area and rapid angiogenesis are of vital importance to cardiac repair after myocardial infarction. The establishment of artificial collateral circulation significantly improves the survival rate of AMI patients, by enhancing both the blood supply to the infarcted area and the viability of myocardial cells [11, 12]. Recent studies demonstrated that promoting angiogenesis in ischemic areas is effective for the treatment of ischemic diseases. Therapeutic angiogenesis, which involves inducing local reangiogenesis in ischemic areas and forms a compensatory collateral circulation, promoting the opening of new blood vessels and self-healing of related cells, can effectively alleviate myocardial ischemia.

A large number of studies demonstrated that miRNAs play regulatory roles in cardiovascular diseases. For example, antagomiRs or recombinant adenoviruses targeting miR-24 improve the recovery of cardiac function in myocardial infarction mice, by modulating the pathway involving endothelial nitric oxide synthase, the endothelial cell transcription factor GATA-2, and the serine/threonine protein kinase PAK4 [13]. In addition, Kim et al. [10] found that the expression of miR-101 in vascular endothelial cells is markedly increased under hypoxic conditions. miRNA-101 promotes endothelial cell growth via the Cul3/Nrf2/heme oxygenase (HO-1)/VEGF pathway, indicating that it plays an important role in the regulation of angiogenesis. During cell and tissue adaptation to hypoxia, genes involved in angiogenesis, cell proliferation, and glucose metabolism are activated. In eukaryotic cells, HIF-1 is the main transcription factor activated by hypoxia and the major regulator of oxygen homeostasis [14].

In the current study, bioinformatics analyses predicted EIF4E3 to be the target of miR-101. EIF4E3 is a eukaryotic translation initiation factor that competitively inhibits the expression of EIF4E1, an important vascular stimulating factor in angiogenesis. By attenuating the activation of the HIF-1 signaling pathway, EIF4E3 inhibits the formation and development of newly born capillaries, subsequently obstructing angiogenesis [15, 16]. EIF4E3 is downregulated in rapidly proliferating tumor cells [17], whereas EIF4E1 (formerly known as EIF4E) is abundantly expressed in tumor cells [18, 19], although it is also present in most cells and plays an important role in angiogenesis. Multiple studies have shown that inhibiting EIF4E can inhibit cell proliferation and angiogenesis [20, 21]. Osborne et al. found that EIF4E3 and EIF4E1 could be integrated into the m7G-cap structure of mRNAs, and that these factors exhibited a competitive relationship. Therefore, EIF4E3 can inhibit the ability of EIF4E1 to promote cell survival and proliferation. In addition, EIF4E3 can also affect mRNA transfer out of the nucleus, thereby inhibiting cell proliferation [15, 16]. Landon et al. found that MNK1/2 inhibits the phosphorylation of EIF4E1 and promotes the expression of EIF4E3, and proposed that MNK1/2 controls cell proliferation by modulating the levels of these translation initiation factors [22]. EIF4E may bind to the cap structure of the *HIF-1α* mRNA and promote the translation of HIF-1*α* mRNA into HIF-1*α* [23–25]. Apart from EIF4E3, the cytokines regulating EIF4E1 were regulated by EIF4E-BPs [26]. Therefore, the ability of EIF4E1 to promote the translation of RNA could be improved; however, this kind of ability was still limited and would not be enlarged indefinitely.

During the adaptation of cells and tissues to hypoxia, the genes involved in angiogenesis, cell proliferation, and glucose metabolism are activated. In eukaryotic cells, HIF-1 is the main transcription factor activated by hypoxia and the major regulator of oxygen homeostasis [14]. HIF-1 was originally discovered as a transcription factor that regulates the expression of erythropoietin in the blood under hypoxic conditions [27, 28]. There are two different subunits of HIF-1, namely, *α* and *β* [29, 30]. To date, three HIF-*α* isomers have been identified: HIF-1*α*, HIF-2*α* (also known as endothelial PAS protein, and HIF-3*α* inhibiting PAS protein), and HIF-3*α*. The second subtype, HIF-2*α*, is expressed in the endothelial cells, lung, and cartilage, and shares 48% amino acid sequence homology with HIF-1*α*. The third subtype, HIF-3*α*, (also known as PAS inhibition protein), inhibits the binding of HIF-1*α* to DNA [31–33]. HIF-1 participates in multiple angiogenic processes. During capillary formation under hypoxic conditions, HIF-1 can directly or indirectly regulate angiogenic factors and cytokines. Enhancing the activity of HIF-1 is imperative to the treatment of ischemic diseases. As mentioned above, angiogenesis is a multistep process [34]; first, hypoxia and HIF-1 directly stimulate the expression of VEGF and its receptor, and then a new angiogenesis process is initiated. Second, the extracellular matrix is degraded by metalloproteinases, allowing migrating endothelial cells to form tubes. Further, HIF-1*α* enhances the expression of matrix metalloproteinase-2 [35]. Third, integrins *α* and *β*, which are induced by HIF, stimulate the proliferation and adhesion of endothelial cells, and HIF-1*α* controls the growth of endothelial cells. The final step of angiogenesis is vascular maturation, which involves the recruitment of vascular sustentacular cells and the formation of the basement membrane.

The treatment goal of angiogenesis is achieved by stimulating the formation of new capillaries via the regulation of angiogenic growth factors. A variety of angiogenic growth factors, such as vascular growth factor (VEGF), placental growth factor, fibroblast growth factor, and platelet-derived growth factor, have been used in *in vitro* studies and preclinical and clinical trials [36–39]. Of these, VEGF may the most important growth factor involved in angiogenesis. The mammalian genome encodes five members of the VEGF family that regulate angiogenesis and lymphangiogenesis, namely, VEGF-A (also known as VEGF), PlGF, VEGF-B, VEGF-C, and VEGF-D [40–43]. In particular, VEGF-A plays a key role in the early stages of angiogenesis. Homozygous and heterozygous *VEGF-A* knockout mice have an embryonic lethal phenotype. Immaturity of the vascular system suggested that the local embryonic vascular concentration is appropriate, and the maturation of neovascularization can be promoted [44, 45]. VEGF-A, VEGF-B, VEGF-C, and VEGF-D are the main factors involved in angiogenesis [46]. Budding and proliferation of new blood vessels are complex processes that involve a wide range of angiogenic factors and their receptors. Under hypoxic conditions, HIF-1 is the main factor upregulating VEGF expression [47, 48]. VEGF induces the expression of FMS-related tyrosine kinase and kinase-binding domain receptors, through paracrine mechanisms [49, 50]. In addition, VEGF specifically and strongly upregulates expression of the plasminogen activator, degrades the basement membrane and matrix of small vessels, increases the permeability of the blood vessels for macromolecules, and promotes the extension, reproduction, migration (through the basement membrane), adherence, and alignment of endothelial cells under the function of chemotactic factors, resulting in the production of open tube-like structures [51–53]. These buds then fuse to form annular vascular branches, containing three-dimensional tubular structures. Perivascular cells constitute the main vascular structure and enable maturation of the collateral circulation, thereby forming a smooth pathway, allowing self-bridging, aiding blood circulation, and alleviating myocardial ischemia [54, 55]. In conclusion, various *in vitro* and *in vivo* studies have demonstrated that VEGF promotes angiogenesis [56, 57].

miR-101 inhibits angiogenesis by targeting enhancer of zeste homolog 2 in tumor tissues [58, 59].Contrarily, Kim et al. found that miR-101 upregulates HO-1 and VEGF by targeting the Cullin 3 protein under hypoxic conditions, thereby promoting angiogenesis [10]. There are similar contradictory reports of the effects of miR-20a, a member of the miR-17-92 cluster, on angiogenesis. Inhibition of miR-20a reportedly promotes [60, 61] or inhibits [62] endothelial cell proliferation and migration. The aim of the current study was to investigate the ability of miR-101 to promote angiogenesis under hypoxic conditions. We found that transfection of HUVECs with agomiR-101 or EIF4E3 siRNA effectively inhibited expression of EIF4E3, and upregulated the HIF-1*α* signaling pathway. VEGF-A expression was higher in cells expressing a combination of agomiR-101 and the EIF4E3-specific siRNA than in those expressing the EIF4E3-specific siRNA alone, suggesting that miR-101 may also promote angiogenesis via other pathways, which is consistent with results from the study by Kim et al. [10].

The EIF4E family of eukaryotic translation initiation factors comprises EIF4E1, EIF4E2, and EIF4E3. Previous studies demonstrated that EIF4E1 plays an important role in the translation of HIF-1*α* [24, 25], promotes transcription of the *HIF-1α* gene, activates the HIF-1 signaling pathway, stimulates the secretion of VEGF-A, and promotes angiogenesis [63]. EIF4E3 competitively inhibits the expression of EIF4E1 [15, 16]. Moreover, EIF4E1 promotes the transcription of HIF-1*α* mRNA into HIF-1*α* [25], which may play an important role in angiogenesis. Therefore, by inhibiting the expression of EIF4E3 and stimulating the HIF-1*α* signaling pathway, thereby promoting the formation and development of new capillaries, miR-101 plays an important role in stimulating the repair and regeneration of the blood vessels. EIF4E3 was identified as a potential target of miR-101 using various bioinformatics packages. Subsequently, by using a luciferase reporter fused to the 3′-UTR of the *EIF4E3* gene, we verified that miR-101 could directly regulate EIF4E3. In a HUVEC model of AMI, overexpression of miR-101 inhibited the expression of EIF4E3, thereby promoting HIF-1*α* expression and stimulating an increase in VEGF-A levels. Furthermore, in EdU, Transwell, and angiogenesis experiments, overexpression of miR-101 effectively enhanced endothelial cell proliferation, migration, and angiogenesis. These findings support our hypothesis that inhibition of EIF4E3 by miR-101 promotes the expression of EIF4E1, thereby enhancing activation of the HIF-1 signaling pathway, stimulating VEGF-A secretion, and promoting the proliferation, differentiation, and migration of endothelial cells. This assumption was further verified using a C57BL/6 AMI mouse model, established by ligating the left anterior descending coronary artery. The mice were then infected with a recombinant adenovirus vector Adv-miR-101. In animal experiments, miR-101 effectively promoted the proliferation of vascular endothelial cells, increased the vascular density after AMI, and improved the ejection fraction, as well as the left ventricular short-axis systolic rate, left ventricular end-systolic diameter, and recovery of cardiac pumping function. However, the recovered myocardial function in mice in the experimental group differed from that of the sham-operated mice. These results indicate that miR-101 can effectively promote angiogenesis and cardiac function recovery in myocardial infarction areas in C57BL/6 mice; however, further studies are needed to confirm that miR-101 can be used to achieve complete recovery of cardiac function following AMI. Kim et al. [10] reported that miR-101 plays an important role in the repair and regeneration of the blood vessels during ischemia using a C57BL/6 mouse hindlimb ischemia model. Kim's group found that miR-101 inhibits the expression of Cullin 3 and activates the expression of HO-1, VEGF, eNOS, and other angiogenesis-related factors, thus reversing ischemia-reperfusion injury. Moreover, there are also contradictory reports of the effects of miR-20a, a member of the miR-17-92 cluster, on angiogenesis. Inhibition of miR-20a reportedly promotes [60, 61] or inhibits [62] endothelial cell proliferation and migration. To the best of our knowledge, the current study is the first report that miR-101 plays a role in AMI, although we recognize that additional experiments are required to further our findings.

To summarize, in the present study, miR-101 expression in HUVECs was increased under hypoxic conditions, which is consistent with the results from Kim et al. [10]. These results may provide new insight for studying the pathogenesis of AMI and may provide novel targets for the diagnosis and treatment of this condition.

## Figures and Tables

**Table 1:** Various parameters of cardiac function in mice by M type ultrasound.

	AMI+Adv-miR-101	AMI+Adv-null	Sham+Adv-null
Interventricular septal diameter (mm)	0.80 ± 0.08	0.84 ± 0.07	0.83 ± 0.06
Left ventricular end-diastolic diameter (mm)	2.69 ± 0.48	2.87 ± 0.33^∗∗^	2.25 ± 0.12
Left ventricular posterior wall diameter (mm)	0.98 ± 0.29	0.98 ± 0.16	0.79 ± 0.21
Left ventricular end-systolic diameter (mm)	1.45 ± 0.27^∗∗^^,#^	1.9 ± 0.22^∗∗^	0.87 ± 0.07
Left ventricular end-diastolic volume (ml)	0.06 ± 0.02^∗^	0.06 ± 0.02^∗∗^	0.03 ± 0.01
LV ejection fraction (%)	82.86 ± 4.77^∗∗^^,##^	69.11 ± 9.81^∗∗^	93.89 ± 1.08
Fractional shortening (%)	45.69 ± 5.11^∗∗^^,##^	33.72 ± 6.41^∗∗^	61.43 ± 2.17
Stroke volume (ml)	0.05 ± 0.02	0.05 ± 0.02	0.03 ± 0.01

^∗^ *P* < 0.05 vs. Sham+Adv-null. ^∗∗^*P* < 0.01 vs. Sham+Adv-null. ^#^*P* < 0.05 vs. AMI+Adv-null. ^##^*P* < 0.01 vs. AMI+Adv-null.

## Data Availability

The data used to support the findings of this study are included within the article.
